# A Review of the Progress in the Microbial Biosynthesis of Prenylated Aromatic Compounds

**DOI:** 10.3390/molecules30193931

**Published:** 2025-09-30

**Authors:** Min Tang, Wanze Zhang, Yanjie Tian, Jianjun Qiao, Xiaobing Li, Weiguo Li, Qinggele Caiyin

**Affiliations:** 1 State Key Laboratory of Synthetic Biology, Tianjin University, Tianjin 300072, China; tm17860747229@163.com (M.T.);; 2 Frontiers Science Center for Synthetic Biology (Ministry of Education), Tianjin University, Tianjin 300072, China; 3 School of Synthetic Biology and Biomanufacturing, Tianjin University, Tianjin 300072, China; 4 Zhejiang Institute, Tianjin University (Shaoxing), Shaoxing 312300, China; 5 College of Chemical Engineering, Shijiazhuang University, Shijiazhuang 050035, China

**Keywords:** prenylated aromatic compounds, biosynthesis, metabolic engineering, biological activities, microbial cell factories

## Abstract

Prenylated aromatic compounds (PACs) are widely distributed in nature and have important applications in medicine, cosmetics, and food due to their antioxidant, anticancer, and anti-inflammatory activities as well as role in the prevention of neurological diseases. Traditional methods of PAC production such as plant extraction and chemical synthesis remain constrained by the low content of these compounds in plants and the complexity of the chemical processes. PACs are synthesized from aromatic compound receptors and prenyl side chain donors, which are in turn synthesized via the shikimate pathway and 2-C-methyl-D-erythritol-4-phosphate/mevalonic acid pathways, respectively. Increasing exploration and research on prenyltransferases (PTs), the key enzymes involved in PAC biosynthesis, have facilitated the emergence of microbial synthesis of PACs as a promising alternative to industrial production. The microbial biosynthesis of PACs is summarized herein, mainly from the perspective of screening and modification of the key enzymes PTs, selection of suitable host systems, and engineering the modification of microbial cell factories to enhance the yields of PACs. The future prospects and challenges of PAC biosynthesis are also discussed.

## 1. Introduction

Aromatic compounds such as L-tyrosine, L-phenylalanine, L-tryptophan, and their derivatives, including coumaric acid, naringenin, and violacein, have been widely used as drugs and food additives; these applications are attributable to their biological activities, which include anticancer and antibacterial activities as well as the prevention of hypertension and arteriosclerosis [1]. The aromatic compounds in plants are mainly present in derivatized forms [2]. Among these, the prenyl modification enhances the lipophilicity of aromatic compounds. This not only increases the bioavailability of aromatic compounds but also enhances their biological activities, including their antioxidant and anti-inflammatory efficacy as well as their ability to prevent neurological diseases. Furthermore, their anticancer activities have been extensively studied in recent years [3,4,5,6,7]. To date, scientists have isolated >1000 prenylated aromatic compounds (PACs) from plants; these include primary metabolites such as ubiquinone and menadione as well as secondary metabolites such as prenyl flavonoids, xanthohumol, and α-tocopherol (vitamin E) [8]. Among these, the prenylated flavonoids account for the largest proportion [9].

PACs are diverse in nature but have a relatively limited distribution and are typically present in low concentrations; they are primarily found in plants of the families Leguminosae, Rutaceae, Cannabaceae, Umbelliferae, Euphorbiaceae, Guttiferae, and Moraceae [2,10]. For instance, xanthohumol is the most common prenylated flavonoid in hops, but constitutes only 1% of its dry weight. Similarly, 8-prenylnaringenin (8-PN) is also found in dried hops, albeit at an even lower concentration of just 0.1% [11]. The extraction of PACs from plants is affected not only by the low PAC content, but also by climate and seasonal changes. Chemical synthesis is usually an effective means of producing high value-added compounds. However, the complex structure of PACs and the expensive and toxic nature of the raw materials render it challenging to achieve environmentally friendly and cost-effective synthesis of these compounds. [2,12,13] Therefore, the development of green and efficient methods of PAC synthesis is of great importance.

Microbial production of PACs presents us with an alternative approach that offers the advantages of reduced pollution, fast cycle, and cost efficiency [13,14,15]. PACs are composed of aromatic compounds framework linked to prenyl side chains, typically dimethylallyl, geranyl, farnesyl, or longer prenyl chains [2,16,17]. The biosynthesis of aromatic compounds occurs via the shikimate pathway, while the prenyl side chains are synthesized via the 2-C-methyl-D-erythritol-4-phosphate (MEP) or mevalonic acid (MVA) pathways. In model chassis strains such as *Escherichia coli*, *Saccharomyces cerevisiae*, and *Bacillus subtilis*, the MEP/MVA pathways are coupled with the activity of prenyltransferases (PTs) to eventually produce PACs [18]. To date, the synthesis of several PACs such as tocotrienols, xanthohumol, and prenylated resveratrol in engineered strains has been synthesized using metabolic engineering and synthetic biology approaches. The current article primarily reviews the progress in the microbial production of PACs. Recent advances in the screening and modification of key enzymes such as PTs, selection of host strains, and the engineering of microbial cell factories to enhance the production of PACs have been introduced herein. Additionally, current challenges and prospects for future development in this field are discussed.

## 2. Biological Activities of PACs

Aromatic compounds inherently possess diverse biological activities. The introduction of a prenyl side chain enhances the lipophilicity of these compounds, increasing their affinity for biological membranes and thereby expanding the range of biological activities of the PACs (Table 1) [8]. These activities include antioxidant, anti-inflammatory, anticancer, and estrogenic effects, as well as protective functions against diabetes-associated complications and neurodegenerative diseases such as Parkinson’s disease and Alzheimer’s disease [19,20,21,22].

Neurodegenerative diseases are among the chronic diseases caused by oxidative stress, and PACs have demonstrated considerable potential for the alleviation and treatment of these diseases. Alzheimer’s disease is one of the most common neurodegenerative disorders and has been extensively studied in animal models. Icariin (ICA) has been shown to reduce endoplasmic reticulum (ER) stress and apoptosis in APP/PS1 transgenic mice by inhibiting the protein kinase R -like endoplasmic reticulum kinase/eukaryotic initiation factor-2α (PERK/eIF2α) pathway, suggesting that ICA may be a promising candidate for the treatment of Alzheimer’s disease [23]. PACs have also been shown to exhibit antidiabetic and antiobesity activities. PACs extracted from licorice can exert antidiabetic effects via the inhibition of protein-tyrosine phosphatase1B (PTP1B) and alpha (α)-glucosidase [24]. Tocotrienols can lower the levels of total cholesterol, low-density lipoprotein cholesterol, and nonhigh-density lipoprotein cholesterol in the blood by inhibiting the activity of 3-hydroxy-3-methylglutaryl-CoA reductase, regulating the genes encoding factor-related apoptosis, sterol regulatory element-binding protein, stearoyl-CoA desaturase 1 and carnitine palmitoyltransferase 1A involved in lipid synthesis, alleviating oxidative stress, and degrading apolipoprotein B [25]. PACs hold considerable potential in the fields of nutraceuticals and pharmaceuticals and continue to garner the attention of clinical researchers. The biological activities of PACs are summarized in the subsequent sections.

### 2.1. Antioxidant Activity

Among the aromatic compounds, polyphenols exhibit antioxidant activity and aid in the elimination or mitigation of the effects of oxidative stress; moreover, prenylation can improve the antioxidant activity to a certain extent. In vitro experiments aimed at evaluating the 1,1-Diphenyl-2-picrylhydrazyl (DPPH) radical scavenging activity of the stilbene compound resveratrol revealed a half maximal inhibitory concentration (IC_50_) of 282.86 ± 12.2 µM, while its prenyl-substituted counterpart 4-C-geranyl resveratrol exhibited an IC_50_ value of 28.09 ± 3.1 µM. This indicates that the DPPH radical scavenging activity of 4-C-geranyl resveratrol is significantly higher compared to that of resveratrol [26]. An analysis of the antioxidant mechanism of pentenyl flavonoids using quantum chemical calculations showed that the addition of prenyl groups can improve the antioxidant capacity of flavonoids. Boulebd evaluated the antiradical mechanisms of 8-prenyldaidzein, licoflavone, and erysubin F (Ery) using hydrogen atom transfer, sequential electron transfer proton transfer, and sequential proton loss electron transfer mechanisms using quantum chemical calculations [27]. Molecular docking analyses of these three compounds with xanthine oxidase (XO) and inducible nitric oxide synthase (iNOS) were subsequently performed. These compounds were found to interact with key residues Glu802 and Arg880 of XO via hydrogen bonds and/or hydrophobic interactions, and with the catalytic residues of iNOS via hydrophobic interactions. The prenyl substituents played a significant role in the binding modes. For instance, the prenyl group of Ery interacted with residues Ala910, Ala1078, Leu1014 and Val1011 of XO, and formed favorable interactions with heme, Met374 and Arg381 of iNOS, demonstrating that prenyl modification positively influences antioxidant activity [27]. A variety of prenyl polyphenols have hitherto been demonstrated to exhibit antioxidant activity. For example, vitamin E has been validated to possess strong antioxidant activity under both in vivo and in vitro conditions. In particular, the vitamin E localized in cell membranes can participate in lipid peroxidation of the membrane. When the cell membranes are attacked by ROS, lipid peroxyl radicals (LOO·) are generated; α-tocopherol can transfer hydrogen ions to LOO· to eliminate the peroxyl radicals and reduce oxidative stress. Each molecule of α-tocopherol can simultaneously eliminate two LOO radicals [28].

Prenylated chalcones such as xanthohumol, isobavachalcone and licochalcone A as well as prenylated flavonoids, including artocarpin, kushenol C and glyasperin A, have been reported to possess antioxidant activity [29]. However, prenyl modifications do not enhance the antioxidant activities of all polyphenols; examples of such compounds include artocarpin, cycloheterophyllin diacetate, artocarpetin and certain other prenyl-modified compounds [30]. Although the effect of prenylation varies with the different polyphenols, most modified polyphenols exhibit an enhancement in antioxidant activities. Oxidative stress plays a critical role in the development of various chronic diseases. The antioxidant effects of PACs can reduce the production of free radicals and ROS, thereby diminishing oxidative stress–associated stimulation of cells and subsequently reducing the progression of chronic diseases [19,20,22].

### 2.2. Anticancer Activity

PACs such as xanthohumol and icaritin exhibit extensive anticancer activities, which have been studied in detail. Icaritin is a prenylated flavonoid extracted from *Epimedium* spp. of the plant family Berberidaceae [31]. It is considered as an effective anticancer agent against various types of cancer, particularly hepatocellular carcinoma (HCC) [6]. The use of icaritin in conjunction with other drugs for the treatment of patients with liver cancer in China is presently under investigation in phase Ⅲ clinical trials [7]. Icaritin reportedly binds to cyclin-dependent kinase 2 (CDK2), inhibiting its expression and activity; this leads to a significant reduction in the phosphorylation of retinoblastoma protein, forkhead box O1, and P27Kip1 (P27), ultimately resulting in cell cycle arrest and increased apoptosis. Moreover, icaritin reduces the degradation of P27 and promotes its expression, forming a positive feedback loop that significantly enhances the inhibitory effect of P27 on CDK2 [32]. In addition, icaritin can induce pyroptosis in the cancer cells of patients with HCC, thereby modulating the tumor immune-microenvironment, promoting the release of inflammatory cytokines and the transformation of macrophages into a proinflammatory phenotype, and inhibiting tumor growth [33].

Xanthohumol, which is found in hops, exhibits antitumor activities against breast, liver, and colorectal cancer [3,4]. Xanthohumol can promote the binding between S-phase kinase-associated protein 2 (Skp2) and cadherin 1 to inhibit the Skp2/protein kinase B/hexokinase 2 signaling pathway, exhibiting inhibitory effects against ovarian cancer [4]. Kuwanon C, which is found in mulberry, interacts with mitochondrial and ER membranes to significantly induce ROS production, disrupt normal organelle membrane structure, inhibit cell cycle progression, and stimulate apoptotic signaling pathways, ultimately leading to the death of HeLa tumor cells [34]. Considering that several compounds such as icaritin and xanthohumol have demonstrated anticancer activities, PACs have great potential to be applied for the development of anticancer drugs.

### 2.3. Anti-Inflammatory Activity

Inflammation is a result of the complex interplay between genetic/environmental factors and immune dysregulation, leading to the development of various diseases such as inflammatory bowel disease (IBD), osteoarthritis (OA), and gout [35]. Numerous PACs have been reported to exhibit anti-inflammatory activities via the modulation of various inflammatory mediators such as interleukin (IL)-1β, tumor necrosis factor-α (TNF-α), IL-6, nitric oxide (NO), and nuclear factor kappa–light chain enhancer of activated B cells (NF-κB) [30]. Vitamin E is known to inhibit the synthesis of prostaglandin E in macrophages by reducing the activity of cyclooxygenase (COX-2) and decreasing the production of proinflammatory cytokines such as IL-1β, IL-6 and TNF-α. It also inhibits the production of IL-2, IL-17, and the proinflammatory chemokine IL-8 to exert anti-inflammatory effects, thus playing an important role in the treatment of IBD [36]. Licochalcone A from licorice was found to exert an anti-inflammatory effect on human osteoarthritic chondrocytes stimulated with IL-1β. As detailed in Zhao et al., the anti-inflammatory activities of 10 PACs extracted from licorice were evaluated using IL-1β-stimulated mouse primary chondrocytes. Among these PACs, glycyuralin Q was found to inhibit NO production, iNOS upregulation, and activation of the NF-κB signaling pathway, highlighting its potential for use as a therapeutic agent for OA [37]. In addition, the prenylated isoflavone derivatives ficucaricones A–D from *Ficus carica* significantly inhibited NO production, with IC_50_ values ranging from 0.89 ± 0.05 to 3.29 ± 0.12 µM, which are lower than that of hydrocortisone (3.68 ± 0.16 µM) [38]. The prenylated coumarins from *Artocarpus heterophyllus* were also found to exhibit notable NO inhibitory activity, with IC_50_ values ranging from 0.58 ± 0.06 to 6.29 ± 0.12 µM [39].

**Table 1:** The Bioactivity of Partial PACs.

**Prenylated Aromatic** **Compounds**	**D** **isease**	**Research**	**Activity**	**Reference**
Artepillin C	Colorectal cancer	Wistar rats In vivo	Anticancer	[40]
	Gastric ulcer	Rats In vivo	Antioxidant	[41]
	SARS-CoV-2	Vero cells and tonsil fragments In vitro	Antiviral Anti-inflammatory	[42]
8-Prenylnaringenin	Colon cancer	Colon cancer HCT-116 cells In vitro	Anticancer	[43]
	Osteopenia	Postmenopausal and osteopenic women In vivo	Phytoestrogen	[44]
7-Geranyloxycoumarin	Gastric adenocarcinoma	MKN45 cells In vitro	Anticancer	[45]
	Chronic inflammation	Male rats In vivo	Anti-inflammatory	[46]
	Chronic obstructive pulmonary disease	Male C57BL/6 mice In vivo	Anti-inflammatory Antioxidant	[47]
4-Geranyl resveratrol	Inflammation	Chemical reagent detection	Anti-inflammatory	[26]
	Cancer	Human hepatoma cancer cells Human breast cancer cells In vitro	Anticancer	[5]
Xanthohumol	Various cancers, such as breast cancer and liver cancer	In vitro	Anticancer	[4,48]
	Neurodegenerative Diseases	In vitro and In animal models	Anti-inflammatory Antioxidant	[49]
	Colitis	Male C57BL/6 mice In vivo	Anti-inflammatory	[50]
Icaritin	Hepatocellular carcinoma	Phase clinical trials	Anticancer	[7]
	Neuroinflammation	Male SD rats	Anti-inflammatory	[51]
Vitamin E	Lung cancer	Chemical reagent detection and Wi3-38 and A549 cells In vitro	Anticancer	[52]
	Neurodegenerative disorders	Computer simulation	Antioxidant	[53]
Kuwanon C	Cervical cancer	HeLa cells In vitro	Anticancer	[34]

## 3. Biosynthesis of PACs

The pathway of PAC biosynthesis is divided into three parts, which include the MEP/MVA pathway for the synthesis of the prenyl side chain donor, the shikimate pathway for the synthesis of the aromatic compound receptors, and the prenylation reaction. The prenylation of aromatic compounds is achieved by the addition of the prenyl side chain donors to aromatic compound receptors via the activity of PTs.

### 3.1. Shikimate Pathway

**Figure 1:**
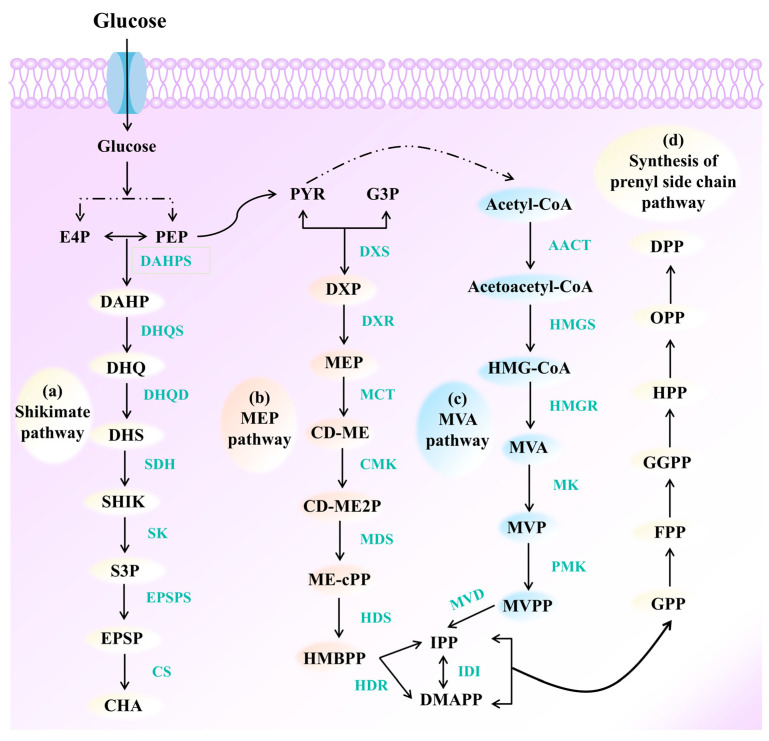
The upstream pathways for the biosynthesis of PACs. (**a**) The shikimate pathway for the synthesis of aromatic receptor precursors. The intermediaries of this pathway are PEP, E4P, DAHP, DHQ, DHS, SHIK, S3P, EPSP, CHA. The enzymes involved in the pathway include DAHP synthase (DAHPS), DHQ synthase (DHQS), DHQ dehydratase/shikimate dehydrogenase (DHQD/SDH), SK, EPSP synthase (EPSPS), chorismate synthase (CS). (**b**,**c**) are two pathways for the synthesis of IPP and DMAPP. (b) MEP pathway. The intermediaries of MEP pathway are G3P, PYR, DXP, MEP, CD-ME, 2-phospho-4-(cytidine 5′-diphospho)2-C-methyl-D-erythritol (CD-ME2P), 2-C-methyl-D-erythritol 2,4-cyclodiphosphate (ME-cPP), and HMBPP. The enzymes involved in the MEP pathway include DXS, DXR, MCT, CMK, MDS, HDS and HDR. (**c**) MVA pathway. The intermediaries of the MVA pathway are acetyl-CoA, acetoacetyl-CoA, HMG-CoA, MVA, 5-phosphomevalonate (MVP), and 5-diphosphomevalonate (MVPP). The enzymes involved in the MVA pathway include AACT, HMGS, HMGR, MK and PMK, MVD, and IDI. (**d**) The pathway for synthesizing prenyl side chain donors with IPP and DMAPP as precursors. The prenyl side chain donors include GPP, FPP, GGPP, heptaprenyl diphosphate (HPP), octaprenyl diphosphate (OPP), decaprenyl diphosphate (DPP).

The shikimate pathway involves the generation of the precursor chorismate, followed by a series of enzymatic reactions that generate the aromatic compound receptors. This pathway is found in plants and microorganisms and is initiated at the intersection of two crucial primary metabolic processes, glycolysis and the pentose phosphate pathway. In shikimate pathway, phosphoenolpyruvate (PEP) and erythrose 4-phosphate (E4P) are combined to generate 3-deoxy-D-arabino-heptulosonate 7-phosphate (DAHP). Following multiple steps of oxidation, phosphate elimination, and reductive cascades, the pyranose DAHP is converted to the first carbocyclic intermediate 3-dehydroquinate (DHQ) [54]. The subsequent dehydration of DHQ generates 3-dehydroshikimate (DHS), the third intermediate of the pathway. The reduction in DHS produces shikimate (SHIK), which is a key metabolic intermediate. The intermediate shikimate-3-phosphate (S3P) of the shikimate pathway is then phosphorylated via the activity of shikimate kinase, followed by reaction with an additional unit of PEP to generate 5-enolpyruvate shikimate-3-phosphate (EPSP). The subsequent removal of phosphate from EPSP yields chorismite (CHA). [54] (Figure 1a)

### 3.2. MEP/MVA Pathway

Isopentenyl diphosphate (IPP) and its isomer dimethylallyl diphosphate (DMAPP) are the donors or precursors for the synthesis of the prenyl side chain. The biosynthesis of IPP and DMAPP in microbial cells occurs via the MEP or MVA pathways. The MEP pathway is mainly found in bacteria, while the MVA pathway chiefly occurs in eukaryotes and archaea. [55]

The MEP pathway is initiated upon the condensation of pyruvate (PYR) and glyceraldehyde 3-phosphate (G3P) by the thiamine diphosphate–dependent enzyme 1-deoxy-D-xylulose-5-phosphate synthase (DXS) to generate 1-deoxy-D-xylulose-5-phosphate (DXP). DXP is then reduced via the activity of 1-deoxy-D-xylulose-5-phosphate reductoisomerase (DXR) to yield MEP. MEP is subsequently converted to 4-(cytidine 5′-diphospho)-2-C-methyl-D-erythritol (CD-ME) via the activity of 2-C-methyl-D-erythritol 4-phosphate cytidylyltransferase (MCT) [56]. CD-ME is then converted to 1-hydroxy-2-methyl-2-butenyl 4-diphosphate (HMBPP) following phosphorylation, cyclization, and ring-opening, catalyzed by 4-diphosphocytidyl-2-C-methyl-D-erythritol kinase (CMK), 2-C-methyl-D-erythritol 2, 4-cyclodiphosphate synthase (MDS), and 1-hydroxy-2-methyl-2-butenyl 4-diphosphate synthase (HDS). Unlike the MVA pathway, where DMAPP is generated via the isomerization of IPP, the MEP pathway involves the direct generation of IPP and DMAPP from HMBPP via the activity of 4-hydroxy-3-methylbutenyl diphosphate reductase (HDR) [56] (Figure 1b).

The MVA pathway is initiated upon the condensation of two molecules of acetyl-coenzyme A (acetyl-CoA) via the activity of the enzyme acetyl-CoA acetyltransferase (AACT) to yield acetoacetyl-CoA. Acetoacetyl-CoA is then combined with another molecule of acetyl-CoA via the activity of 3-hydroxy-3-methylglutaryl-CoA synthase (HMGS) to produce 3-hydroxy-3-methylglutaryl-CoA (HMG-CoA) [56]. This compound is subsequently reduced by 3-hydroxy-3-methylglutaryl-CoA reductase (HMGR) to produce mevalonate. Mevalonate undergoes two phosphorylation and one decarboxylation reaction catalyzed by mevalonate kinase (MK), phosphomevalonate kinase (PMK), and mevalonate diphosphate decarboxylase (MVD), respectively, to ultimately generate IPP. The enzyme isopentenyl diphosphate isomerase (IDI) catalyzes the subsequent isomerization of IPP to DMAPP [56] (Figure 1c).

IPP and DMAPP can be produced via both the pathways, albeit with pathway-dependent differences in energy consumption and cofactor usage, which are expected to affect the preferences for the two pathways. For the generation of one molecule of IPP/DMAPP, the MVA pathway consumes 1.5 molecules of glucose and produces four molecules of nicotinamide adenine dinucleotide phosphate (NAD(P)H), with a maximum glucose yield of 25.2% [57]. By contrast, the MEP pathway consumes one molecule of glucose, three molecules of adenosine triphosphate, and two molecules of NAD(P)H. When normalized to glucose consumption, the MEP pathway requires 1.25 molecules of glucose to produce one molecule of IPP/DMAPP, with a maximum glucose yield of 30.2%. The MEP pathway therefore offers a higher theoretical yield compared to that of the MVA pathway, but entails greater cofactor consumption [57]. The synthesized IPP (C5) and DMAPP (C5) were further converted to linear geranyl pyrophosphate (GPP) via head-to-tail condensation reaction and subsequently condensed into prenyl pyrophosphates of different lengths, including farnesyl pyrophosphate (FPP) and geranyl geranyl pyrophosphate (GGPP). This expands the product diversity, allowing them to function as prenyl donors while contributing to a broader range of biochemical compounds [58] (Figure 1d).

### 3.3. Prenylation Reaction

**Figure 2:**
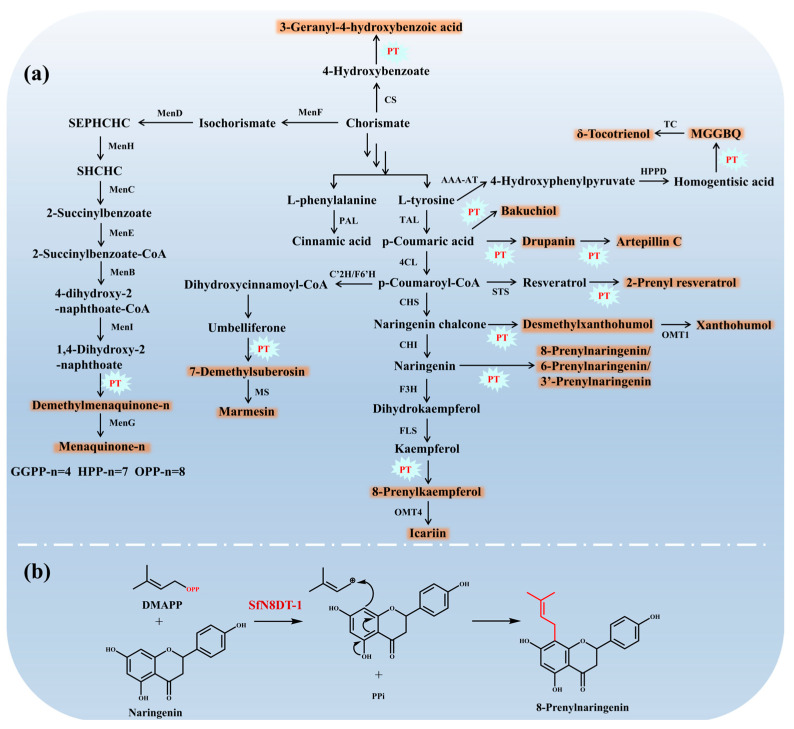
The downstream pathways of synthetic PACs. (**a**) Biosynthetic pathways and enzymes for the heterologous production of PACs. The enzymes involved in the pathways include PT, phenylalanine ammonialyase (PAL), tyrosine ammonialyase (TAL), 4-coumarate-CoA ligase (4CL), chalcone synthase (CHS), chalcone isomerase (CHI), flavanone 3-hydroxylase (F3H), flavonol synthase (FLS), 4’-O-methyltransferase (OMT4), 6’-Omethyltransferase (OMT1), stilbene synthase (STS), aromatic amino acid transaminase (AAA-AT), 4-hydroxyphenylpyruvate dioxygenase (HPPD), tocopherol cyclase (TC), p-coumaroyl-CoA 2’-hydroxylase (C2’H), feruloyl-CoA 6’-hydroxylase (F6’H), marmesin synthase (MS), CS, isochorismate synthase (MenF), 2-succinyl-5-enolpyruvyl6-hydroxy-3-cyclohexene-1-carboxylate synthase (MenD), demethylmenaquinone methyltransferase (MenH), O-succinylbenzoate synthase (MenC), O-succinylbenzoate-CoA ligase (MenE), 1,4-dihydroxy-2-naphthoyl-CoA synthase (MenB), 1,4-dihydroxy-2-naphthoyl-CoA hydrolase (MenI), demethylmenaquinone methyltransferase (MenG). MGGBQ: 2-methyl-6-geranylgeranyl benzoquinol; SEPHCHC: 2-Succinyl-5-enolpyruvyl-6-hydroxy-3-cyclohexene-1-carboxylate; SHCHC: (1R,6R)-2-succinyl-6-hydroxy-2,4-cyclohexadiene-1-carboxylate. (**b**) Example of a prenylation reaction: biosynthesis of 8-prenylnaringenin from DMAPP and naringenin catalyzed by prenyltransferase SfN8DT-1.

**Figure 3:**
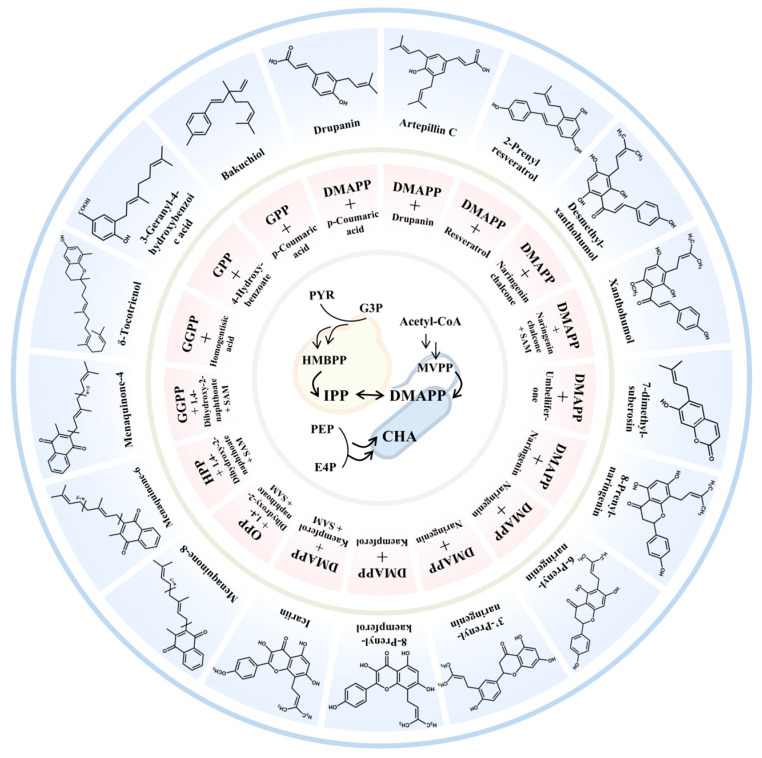
The precursors of the biosynthesis of PACs and the structures of PACs. 7-Dimethylsuberosin is the precursor of marmesin.

Prenylation reaction represents the most critical step in the biosynthesis of PACs and is catalyzed by the key enzymes PTs. PTs exhibit selectivity for specific substrates and can catalyze the Friedel–Crafts alkylation of different prenyl side chains found in various aromatic compounds [59]. Homogentisate phytyltransferase (HPT) is found in plants and catalyzes the reaction between phytyl pyrophosphate and homogentisic acid (HGA) to produce tocopherols. Homogentisate geranylgeranyltransferase (HGGT) catalyzes the reaction between GGPP and HGA to yield tocotrienols. The enzyme 1,4-dihydroxy-2-naphthoate (DHNA) heptaprenyltransferase from *Synechocystis* sp. PCC 6803 (SyMenA) catalyzes the reaction between DHNA and FPP to produce menaquinone-4 (MK-4) [60], while BsMenA from *B. subtilis* catalyzes the reaction between DHNA and hexaprenyl diphosphate to produce menaquinone-7 (MK-7) [61]. The regioselectivity of PTs allows them to catalyze the linking of prenyl side chains to different positions of aromatic compounds [59]. SfN8DT-1 from *Sophora flavescens* catalyzes the linking of DMAPP to C-8 of naringenin to yield 8-PN [62], while AnaPT from *Neosartorya fischeri* catalyzes the binding of DMAPP to the 3′-C of naringenin [63]. Therefore, the substrate and regioselectivity of PTs govern the structural diversity of PACs (Figure 2 and Figure 3).

**Figure 4:**
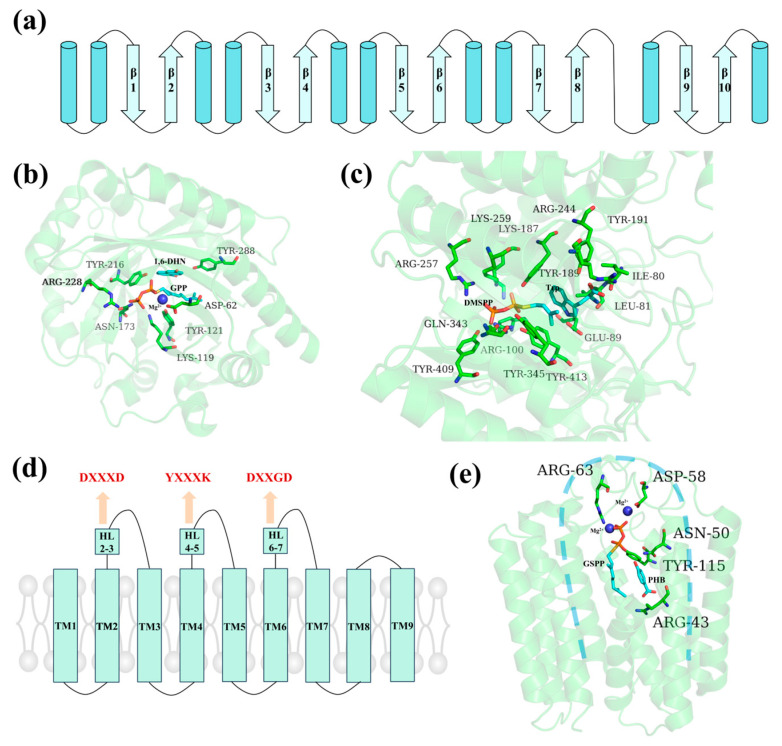
Structural features of PTs. (**a**) The ααββ repeat structure of the sPT-type. Its central barrel is formed by 10 antiparallel β-strands. (**b**) ABBA-type NphB complexed with GPP, Mg^2+^, and 1,6-DHN (PDB: 1ZB6). (**c**) DMATS-type FgaPT2 complexed with L-tryptophan and DMSPP (PDB: 3I4X). (**d**) Membrane topology diagram of UbiA-type aPTs, showing the positions of two conserved Asp-rich motifs (DxxxD and DxxGD) and one conserved YxxxK motif. The structure contains nine transmembrane helices (TM) forming a central cavity, with the C-terminal extensions of TM2, TM4, and TM6 (HL2-3, HL4-5, and HL6-7) constituting a lid region over the cavity. (**e**) Ap UbiA complexed with GSPP, PHB, and Mg^2+^ (PDB: 4OD5).

Extensive research has been carried out on PTs in recent years. PTs can be divided into two types based on their evolutionary origins, structures, and intracellular localizations; these include soluble (sPT-type, including dimethylallyl tryptophan synthase (DMATS) and the cytoplasmic α-β-β-α barrel isopentenyl transferase (ABBA), and intracellular PTs (UbiA-type) (Figure 4) [18,64]. The divalent metal ion-dependent UbiA-type PTs are derived from animals, plants, bacteria, and fungi. They participate in the biosynthesis of various secondary metabolites such as tocopherols, tocotrienols, menaquinones, and prenylated naringenins. Compared to the UbiA-type PTs, the sPT-type PTs found in bacteria and fungi are less involved in the biosynthesis of PACs that share the MEP/MVA and shikimate pathways. The sPT-type proteins feature an αββα PT-barrel structure, formed by a central barrel consisting of 10 antiparallel β-strands surrounded by solvent-exposed α-helices (Figure 4a–c). The UbiA-type proteins do not exhibit any sequence or structural similarities with the sPT-type proteins. Instead, they adopt a counterclockwise-arranged U-shaped topology that forms a large central cavity containing two conserved Asp-rich motifs (DxxxD, and DxxGD) and one conserved YxxxK motif (where x represents any residue) (Figure 4d,e) [65]. The crystal structures of some PTs such as NphB (ABBA) (Figure 4b), FgaPT2 (DMATS) (Figure 4c), and ApUbiA (UbiA) (Figure 4e) are presently available [18,64]. These structural insights have significantly advanced our understanding of the molecular mechanisms underlying the substrate specificity and catalytic activity of PTs.

## 4. Strategies for Enhancing the Biosynthesis of PACs

Currently, the industrial production of PACs primarily relies on extraction from plants or chemical synthesis. For instance, the industrial production of vitamin E is based on a seven-step chemical synthesis process [12], while that of prenylated flavonoids is mainly achieved via extraction from plants [66,67]. Issues associated with high land occupation and long production cycle plague the approach of PAC extraction from plants. By contrast, the chemical synthesis of PACs involves multiple steps and large amounts of organic reagents, resulting in high costs and environmental pollution. The approach of microbial synthesis of PACs presents a highly advantageous alternative. The existence of the MEP/MVA and shikimate pathways in microorganisms and identification of key enzymes (PTs) have laid a solid foundation for the complete synthesis of PACs in microorganisms. To date, several PACs such as tocotrienols, prenylated flavonoids, menaquinones, drupanin, and artepillin C have been successfully synthesized in microorganisms.

### 4.1. Screening and Modification of Key Enzymes for the Synthesis of PACs

**Figure 5:**
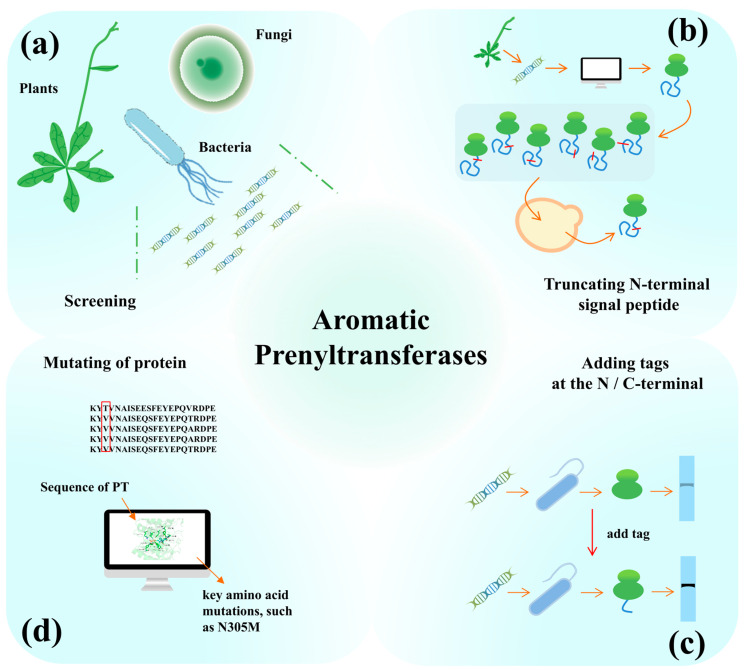
Screening and modification of key enzymes PTs for PAC synthesis. (**a**) Screening PTs from different sources. (**b**) Truncating N-terminal signal peptide. (**c**) Adding tags at the N/C-terminal. (**d**) Mutating of protein.

PTs catalyze the reaction between prenyl side chains and aromatic compounds at a 1:1 molar ratio to yield PACs. This step represents a key rate-limiting step in the PAC biosynthetic pathway. Microbial hosts present different intracellular microenvironments from that of the natural plant hosts, which may lead to misfolding, reduced levels of expression, and diminished catalytic efficiency of plant-derived enzymes in these heterologous environments [68]. Therefore, PTs from various sources were screened to allow selection of enzymes with high catalytic activity and efficiency in microbial hosts (Figure 5a). Based on the National Center for Biotechnology Information database, Guo et al. selected six PTs from different sources to identify the enzymes with catalytic activity; only one of the PTs, SfN8DT-1 derived from *S. flavescens*, demonstrated the ability to catalyze the conversion of naringenin to 8-PN in the heterologous host *S. cerevisiae* [69].

Plant-derived PTs exhibit membrane-binding properties and strict regiospecificity [59], limiting the microbial production of various PACs. PTs from bacteria and fungi, which have been identified as capable of catalyzing the prenylation of aromatic substrates, can therefore be employed as alternatives to plant-derived PTs for the microbial production of PACs [63]. Shen et al. screened 14 PTs from plants and fungi; only HPT from *Synechocystis*. sp. PCC6803 was successfully expressed in *S. cerevisiae* and found to catalyze the transfer of GGPP to HGA to produce the tocotrienol intermediate 2-methyl-6-geranylgeranyl benzoquinol [70]. Isogai et al. screened 11 PTs from actinomycetes, and fungi using in vitro experiments and subsequently validated these PTs in *S. cerevisiae*. Among these, codon-optimized AnaPT, CdpC3PT, and CdpNPT were found to catalyze the transfer of prenyl group to naringenin to yield 3′-prenylnaringenin [63]. Therefore, microorganisms and plants are both important sources for the identification and screening of PTs. Regardless of their origin, however, only around 20 PTs have hitherto been shown to catalyze prenylation reactions in heterologous microbial hosts (Table 2), and the functions/activities of a large number of PTs remain to be validated. An ongoing effort to identify and screen PTs from diverse sources can not only yield PTs of higher efficiency, but also facilitate the biosynthesis of a variety of PACs in heterologous hosts.

Plant-derived PTs function in specific subcellular compartments of plant cells such as chloroplasts [59], and thus have N-terminal signal peptides in their amino acid sequences. When such PTs are expressed in microorganisms that lack the mechanism to remove these compartment-specific signal peptides, the presence of these sequences may hinder the expression of the protein, thereby affecting the activity and function of the PTs in the heterologous host (Figure 5b) [59,70]. Wang et al. demonstrated that an N-terminal truncation of EsPT2 from *Epimedium sagittatum* involving the deletion of 30 amino acids (aa) enhanced the catalytic activity of the protein in *S. cerevisiae*, resulting in a 3.3-fold increase in the production of 8-prenylkaempferol (8-KAE) [31]. In silico signal peptide prediction studies by Chaojie et al. on SfN8DT-1 that was heterologously expressed in *S. cerevisiae* identified a signal peptide side chain of 120 aa. Truncations were then performed at 13 different positions. A truncation length of 62 aa supported the highest activity of SfN8DT-1, resulting in a 290% enhancement in the production of 8-PN [88]. An appropriate truncation of plant-derived PTs typically enhances their enzymatic activity, likely because the deletion of signal peptide sequences facilitates proper folding or improves solubility of the proteins in heterologous hosts [89,90].

Although *E. coli* is an excellent system for the expression of heterologous proteins, drawbacks such as low solubility of the expressed protein remain. The addition of fusion tags to the N-terminus of expressed proteins remains the main method of increasing protein solubility in *E. coli* (Figure 5c) [91]. The common fusion tags employed for protein expression in *E. coli* include thioredoxin A, maltose-binding protein, glutathione S-transferase (GST), small ubiquitin-like modifier (SUMO), and N-utilizing protein (NusA) [91]. Following a 60 aa N-terminal truncation, Zhao et al. sought to further improve the catalytic activity of the truncated EsPT2 by enhancing its solubility in *E. coli* by using the fusion tags GST, NusA, and SUMO. Only the SUMO tag allowed a 2-fold increase in the production of 8-KAE by the truncated EsPT2, while the use of the other two tags decreased it [92]. Although N-terminal fusion tags can enhance the expression of heterologous proteins in *E. coli*, their effectiveness is selective.

The advent of big data tools and the development of artificial intelligence (AI) technology have allowed the emergence of a few AI assisted tools for predicting and designing proteins, AlphaFold being an example. Protein engineering has evolved from directed evolution to rational and de novo design. Unlike rational design, directed evolution is not dependent on the availability of comprehensive information of the protein and its three-dimensional structure [93]. An increasing number of reports on the structures, catalytic sites, and reaction mechanisms of PTs have been witnessed in recent years. These studies reflect the considerable progress made in improving the activity of PTs by altering either the key residues in PTs or the regioselectivity of prenyl side chain donors and aromatic receptors (Figure 5d). Guo et al. carried out mutagenesis of PTs based on homologous sequence alignment and protein structure. Four PTs from *S. flavescens* with >90% sequence similarity were selected for sequence alignment; 12 residues with low conservation were identified and mutated to the corresponding residues of sequence alignment [69]. Among these, the Q12E mutant increased the production of 8-PN by 11.2%. Subsequently, template-free structure prediction of SfN8DT revealed that the Q12E mutation did not alter the main structure of the protein, but rather, caused changes in the side chains. Following the docking of the substrate 2S-naringenin and DMAPP with SfN8DT-Q12E, virtual saturation mutagenesis of these two ligands within a 5-Å range allowed the identification of two key residues P229 and N305. Saturation mutagenesis at these two sites can increase the yield of N305M by 34.1% compared with the non-mutant site [69]. Additionally, the X-ray crystal structure of PTs was analyzed to determine the binding mode and key amino acid residues of the prenyl side chain donor and aromatic receptors with PTs; this will allow the length of the prenyl side chain involved in the prenylation reaction and the regioselectivity of the aromatic compound acceptor to be altered via the mutation of key amino acid residues [18]. Mori et al. mutated the key residue Ala173 (which binds to the prenyl side chain) of the PT TleC from *Streptomyces blastmyceticus* to Met, changing the prenyl side chain from GPP (C10) to DMAPP (C5) [94]. Similarly, the site-directed mutagenesis (G115T) of FtmPT1 from *Aspergillus fumigatus* blocked or inhibited the rotation of the carbocation intermediate, shifting the prenylation position from the conventional C-2 to the reverse C-3 position. While the crystal structures and catalytic mechanisms of PTs have been partially elucidated, most of these studies were focused on fungi-derived PTs. A considerable gap remains in the analysis of plant-derived PTs, which is a critical factor limiting the improvement of PT activity (Figure 5c).

### 4.2. Selection of Suitable Hosts for Enhancing the Production of PACs

At present, the widely employed strains for PAC synthesis include *S. cerevisiae*, *E. coli*, and *B. subtilis*. All of them are commonly used engineered strains with clear genetic backgrounds, among which *S. cerevisiae* has been granted “Generally Recognized as Safe” status by the U.S. Food and Drug Administration, particularly. [13,14,15]. These strains have clear genetic backgrounds and are easily amenable to genetic manipulation (Table 3). Moreover, *E. coli* offers the advantages of fast growth, ability to support high cell-density fermentation, low cost of culture, and the availability of various excellent genetic tools [13]. Gao et al. introduced the MVA pathway in *E. coli*, optimized both the MVA and DHNA pathways via gene overexpression and promoter replacement, and combined membrane engineering to achieve MK-7 yields of 1.35 g/L in a 1 L fermenter, effectively resolving the imbalance between MK-7 production and the biomass of engineered strains of *Bacillus* [95]. Similarly, *S. cerevisiae* is also easy to culture but has higher tolerance for glucose compared to that of *E. coli*. As an ideal eukaryotic host, *S. cerevisiae* can support several post-translational modifications that are important for the expression of certain proteins such as cytochrome P450. It also contains multiple organelles and a complete intracellular membrane system, providing diverse environments for compartmentalized biosynthesis and enabling better expression of membrane-bound proteins than *E. coli* [15,96]. An et al. used LaPT2 from *lupinus albus* to catalyze the synthesis of 8-KAE from DMAPP and kaempferol. Plant-derived PTs often possess transmembrane domains and are naturally localized to plastids. The targeting of LaPT2 to the mitochondria increased the production of 8-KAE by 1.05-fold compared to the levels attained upon the cytosolic expression of the enzyme [97]. To date, the heterologous synthesis of PACs such as tocotrienols, 8-PN, prenylresveratrol, and menaquinone has been achieved in *E. coli* and *S. cerevisiae* [62,70,98].

The traditional Japanese food natto, made from soybean fermented using *B. subtilis*, is known to contain a high concentration of MK-7 [99]. Additionally, the high growth rate of *B. subtilis* supports a shortened fermentation cycle [15]. Therefore, *B. subtilis* is the preferred strain for the fermentative production of menaquinone. By optimizing the composition of the fermentation medium and conditions, MK-7 yields of up to 226 mg/L have been attained in a 3 L bioreactor [100]. Furthermore, the development of genetic manipulation tools has allowed metabolic engineering in *B. subtilis*, enabling the heterologous synthesis of other configurations of menaquinone, such as MK-4, in this strain. For instance, Yuan et al. expressed the PT SyMenA and methyltransferase SyMenG from *Synechocystis* sp. PCC 6803 in *B. subtilis* and achieved MK-4 yields of 145 ± 2.8 mg/L in a 3 L bioreactor via metabolic regulation [60]. In addition to the above three strains, *Lactococcus lactis*, *Bacillus amyloliquefaciens*, and *Komagataella phaffii* have been employed for the production of PACs; however, these have not been as widely employed, given that genetic manipulation in these microorganisms is not as easy as in *E. coli*, *S. cerevisiae*, or *B. subtilis*.

**Table 3:** Engineered Strains and production strategies for PAC production.

**M** **icroorganism**	**Product**	**O** **riginal** **S** **train**	**Strategies**	**Yield**	**Reference**
*Saccharomyces cerevisiae*	δ-Tocotrienol	BY4742	Overexpression of *tHMG1* and *Gppssa* from *Sulfolobus acidocaldarius* Optimization of fermentation medium by response surface methodology	4.10 ± 0.10 mg/L	[101]
	Tocotrienols	BY4742	Screening of PTs Truncation of the N-terminal signal peptides of MPBQMT, TC, and γ-TMT Knockout of *Aro3*, *Aro10*, and *YPL062W* Expression of *Aro4^K229L^*, *Aro7^G141S^*, *TyrC* from *Zymomonas mobilis*, and *CrtE03M* Overexpression of *Tkl1* and *tHMG1* Fermentation using a cold-shock-triggered temperature control system	320 mg/L	[70]
	Tocotrienols	YSM5	Knockout of *MOT3* Overexpression of *CrtE03M* and *POS5* Two-phase fermentation using olive oil Overexpression of the genes of transporters PDR11 and Yol075c	82.68 mg/L	[102]
	δ-Tocotrienol	BY4741	Knockout of *GAL80*, *ROX1*, *DOS2*, *Aro3* and *Aro10* Overexpression of *tHMG1*, *CrtE03M*, *POS5*, *Tkl1* and *PDR1* Expression of *Aro4^K229L^*, *Aro7^G141S^*, and *TyrC* from *Z.mobilis* Overexpression of the coding genes of TC, HPT, and HPPD with a copy number ratio of 2:3:1 Two-phase fermentation using olive oil, 2-HP-β-Cyclodextrin derivatives	211.56 mg/L	[103]
	8-prenylnaringenin	W303-1A-Δcoq2		0.51 ± 0.0693 μg/L	[62]
		IMK393	Overexpression of *tHMG1* Replacing *TSC13* with *MdECR* from *Malus domesticus* Expression of *TAL1* from *Rhodobacter capsulatus*	0.12 mg/L	[72]
		CENPK2-1D	Screening of PTs and N-terminal truncation Identification of key residues through multiple sequence alignment Overexpression of *tHMAG1* and *IDI* Template-free structure prediction of tSfN8DT-1, followed by molecular docking and subsequent mutagenesis	101.40 ± 2.55 mg/L	[69]
	3′-prenylnaringenin	YPH499	Construction of naringenin synthesis pathway Screening promiscuous microbial PTs	1.10 ± 0.0962 μg/L	[63]
	xanthohumol	SY03	Enhancing PT activity through enzyme mining, signal peptide truncation, and increased expression levels Knockout of *ARO10* Expression of *Aro4^K229L^* and *Aro7^G141S^* Overexpression of FPPS mutant gene *ERG20^N127W^* and key rate-limiting MVA genes Downregulation of *ERG20^N127W^* expression Fusion of *IDI* and *HlPT1L_Δ1-86_*	0.14 mg/L	[75]
	icaritin	CEN.PK2-1C	Screening of PTs and GmOMT2 Targeting GmOMT2 to mitochondria or coculturing with *E. coli*	19.7mg/L	[31]
		CEN.PK2-1D	Screening of PTs and methyltransferase, ultimately selecting EkF8PT from *Epimedium koreanum* and MpOMT4 from *Mentha x piperita* Introducing the IUP pathway and overexpressing MVA pathway genes Truncating the N-terminal of EkF8PT Expressing methylenetetrahydrofolate reductase (MTHFR) from *Arabidopsis thaliana* and *MET13* from *S.cerevisiae* Performing rational design on MpOMT4	14.4 mg/L	[76]
		CEN.PK2-1D	Screening and identification of PTs from *E.koreanum* Overexpressing *tHMG1* and *IDI* Truncating the N-terminal disordered region of EkF8DT3 Expressing *GmOMT2* from *Glycine max*	172.0 mg/L	[77]
	3-geranyl-4-hydroxybenzoate acid	WAT11U	Screening of PTs Expression of *ERG20^K197G^* and *UbiC* from *E. coli* Overexpression of *tHMG1*	179.29 mg/L	[79]
	bakuchiol	BY4742	Screening of PTs and N-terminal truncation Overexpression of *ERG20^F96W/N127W^* and *tHMG1* Knockout of *PDS5* and *ARO10* Expression of *Aro4^K229L^*, *Aro7^G141S^* and *FiTAL* Fusion of ERG20^F96W/N127W^ and PcPT07t	9.28 mg/L	[83]
	marmesin	BY4741	Expressing of *Aro4^K229L^*, *Aro7^G141S^*, the genes of L-tyrosine prephenate dehydrogenase from *Zymomonas mobilis*, phosphoenolpyruvate synthase from *E. coli*, tyrosine, ammonia-lyase from *Rhodosporidium toruloides* and coumarin synthase (AtCOSY) from *Arabidopsis thaliana* Direct fusion of p-coumaroyl CoA 2′-hydroxylase from *Peucedanum praeruptorum* and 4-coumaroyl-CoA ligase from *Petroselinum crispum* Truncating the N-terminal signal peptides of PcU6DT (umbelliferone 6-dimethylallyltransferase from *P. crispum*), FcMS (marmesin synthase from *Ficus carica*), and AtCPR1 (CYP450 reductase 1 from *A. thaliana*) Overexpressing *PcU6DT*, *FcMS*, and *AtCOSY*	27.7 mg/L	[104]
*Escherichia coli*	δ-Tocotrienol	DH5α		15 µg/g	[71]
	2-methyl-6-geranylgeranyl-benzoquinol(MGGBQ)	DH5α	Overexpression of *IDI* and *DXS*	1425 µg/g	[105]
	Licoflavanone(C3′-prenylnaringenin)	BL21 (DE3)	Screening PTs Introduction of IUP pathway Optimizing fermentation conditions	537.8 mg/L	[106]
	3geranyl-4-hydroxybenzoic acid (GBA)	Rosetta (DE3)	Introduction of MVA pathway	94.30 mg/L	[78]
	6-prenylnaringenin	BL21 (DE3)	Introduction of the IUP pathway and screening of pathway enzymes Optimization of carbon sources and biotransformation conditions	69.9 mg/L	[73]
	Prenylated stilbenoids	BL21 (DE3)	Optimization of fermentation conditions Replacement of promoters for genes synthesizing acetyl-CoA and malonyl-CoA	68.4 mg/L	[98]
	Menaquinone-8 (MK-8)	JM109	Knockout of *UbiC/A* Overexpression of *MenA*	290 µg/g	[86]
	Menaquinone-7 (MK-7)	G01/ pLE2SK	Expression of *HepPPS* from *B. subtilis* Low expression of *MvaE*, *MvaS*, and *MK*	8.8 mg/L	[85]
		BW25113	Introduction of the MVA pathway and enhanced expression of *IDI* Combinatorial expression of *HepPPS*, *UbiE* from *B. subtilis* and *Men A* from *E. coli* Replacement of the native *MenFDCEB* promoter with the strong inducible *BAD* promoter Enhancement of MK-7 synthesis through membrane engineering	1.35 g/L	[95]
*Bacillus subtilis*	Menaquinone-7 (MK-7)	*B. subtilis* 168	Modular expression Overexpression the coding genes of MenA, DXS, DXR, YacM, YacN and GlpD. Knockout *dhbB*	69.5 ± 2.8 mg/L	[61]
		*B. subtilis* 168	Overexpression the coding genes of MenA, DXS, DXR and IDI	50 mg/L	[107]
		*B. subtilis* 168	Develop the Phr60-Rap60-Spo0A quorum sensing system and utilize this system to dynamically regulate the expression of key enzymes	360 mg/L	[108]
		*B. subtilis* 168	Co-expression of the cell membrane component signal transduction proteins tatAD-CD and menaquinol-cytochrome c reductase qcrA-C	410 mg/L	[109]
	Menaquinone-4 (MK-4)	*B. subtilis* 168	Overexpression the coding genes of MenA, MenG, and CrtE from *Synechocystis* sp. PCC 6803 Knockout of the *hepT*, which catalyzes the conversion of farnesyl diphosphate to heptaprenyl diphosphate Simultaneous overexpression the coding genes of DXS, DXR, and IspD-IspF in the MEP module under the strong promoter *P43* Heterologous expression of the MVA pathway	145 ± 2.8 mg /L	[60]
Other					
*Bacillus amyloliquefaciens*	Menaquinone-7 (MK-7)	*B. amyloliquefaciens* Y-2	Comparison of production capabilities between *Bacillus amyloliquefaciens* W21 and Y-2 strains Comparison of shake flask and static culture Overexpression of *HepS*	273 ± 5.4 µg/g DCW	[110]
*Lactococcus lactis*	Menaquinone	*L*.*lactis* ssp. cremoris MG1363	Co-overexpression of *PreA*, *MenA* and *Mk*	719 ± 33.0 nmol/L	[111]
*Komagataella phaffii*	Artepillin C	*K. phaffii* CBS7435	Expression of *TAL* from *Herpetosiphon aurantiacus* Expression of *Aro4^K229L^* and *Aro7^G141S^* Overexpression of *IDI* and *tHMG1*	12.5 ± 0.9 mg/L	[82]
*Yarrowia lipolytica*	Artepillin C	Po1f	Overexpress the rate-limiting genes of the MVA pathway, introduce the *MvaE* and *MvaS* from *Enterococcus faecalis* and the *MK* from *Methanosarcina mazei*, and reduce the strength of the *ERG9* promoter Overexpress the genes of *TAL*, *ARO4^K221L^* and *ARO7^G139S^* Construct diploid strains	7.45 mg/L	[112]
	8-prenylnaringenin	Po1f	Overexpress the genes responsible for naringenin synthesis Overexpress the genes involved in the synthesis of acetyl-CoA and malonyl-CoA Overexpress *ARO4^K221L^ and ARO7^G139S^* Construct diploid strains	4.36 mg/L	[112]

### 4.3. Modification of Engineered Strains to Enhance PAC Production

#### 4.3.1. Metabolic Engineering of PAC Synthesis Pathway

**Figure 6:**
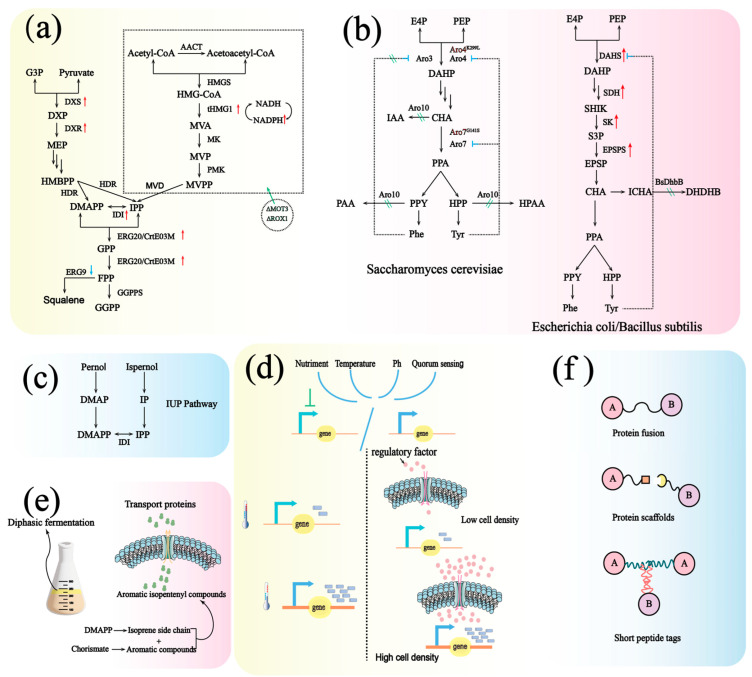
Engineering modification of microorganisms to enhance the synthesis of PACs. (**a**) Modification of the MEP/MVA pathway. (**b**) Modification of the shikimate pathway. (**c**) IUP pathway. (**d**) Dynamic regulation of the pathway. (**e**) Efflux engineering of PACs. (**f**) Protein fusion engineering.

The synthesis of PACs can be summarized as the PT-catalyzed prenylation of the products of the shikimate pathway using the products of the MEP/MVA pathway. An enhanced production of PACs can be achieved through multiple nodes in the synthesis pathway (Table 3). The precursor flux can be improved by overexpressing the rate-limiting enzymes, carrying out knock outs or downregulation of genes of competing pathways, relieving feedback inhibition, and introducing highly active heterologous enzymes and heterologous synthesis pathways of various precursors. For the prenyl side chain, overexpression of the rate-limiting enzymes of the MEP (DXS and DXR) or MVA (HMGR) pathways in *E. coli*, downregulation of the squalene synthase ERG9 (with role in a competing pathway), and knock out of the MVA pathway transcription inhibitor MOT3 and ROX1 in *S. cerevisiae* can enhance the synthesis flux of DMAPP and IPP [113]. Endogenous farnesyltranstransferase BTS1 in yeast, due to its low catalytic activity, is the main rate-limiting enzyme for the synthesis of GGPP from DMAPP and IPP; the synthesis flux to GGPP can be increased by introducing wild-type geranylgeranyl diphosphate synthase CrtE (derived from *Xanthophyllomyces dendrorhous*), its mutant CrtE03M, or the mutant FPS^F112A^ (capable of directly generating GGPP from IPP and DMAPP) of the enzyme derived from *Gallus gallus* (Figure 6a) [113,114].

Although both the MVA and MEP pathways can synthesize IPP and DMAPP, the MEP pathway exhibits higher carbon utilization efficiency, while the MVA pathway consumes fewer cofactors and ATP [57]. Although each pathway has distinct advantages, with superior ones being selectable for engineering, the host’s inherent pathway is usually prioritized in practice. However, its performance is frequently constrained by intracellular regulatory mechanisms, limiting further optimization. To overcome this, heterologous MVA or MEP pathways have been successfully introduced in various studies. For instance, the introduction of a heterologous MVA pathway into *E. coli* created a synergistic effect with the native MEP pathway. This strategy increased isoprene production by 20-fold and 3-fold, respectively, and enhanced carbon flux by 4.8-fold and 1.5-fold compared to overexpressing either pathway alone [115]. Similarly, Gao et al. achieved a 22-fold increase in MK-7 titer by introducing a heterologous MVA pathway along with *B. subtilis*-derived HepPPS (BsHepPPS) [85].

In recent years, an orthogonal isopentenol utilization pathway independent of the endogenous MVA/MEP pathways has been elucidated and applied for the synthesis of prenyl side chains. This pathway synthesizes IPP and DMAPP via the two-step phosphorylation of prenol/isoprenol in microorganisms, which can increase the flux of DMAPP and IPP without imposing additional cellular burden. However, the intrinsic toxicity of prenol and isoprenol to cells limits their widespread application (Figure 6b) [116].

The expression of feedback inhibition–resistant mutants of chorismate mutase (Aro7^G141S^) and 3-deoxy-D-arabinoheptulosonate-7-phosphate synthase (Aro4^K229L^) as well as generation of knockouts of indolepyruvate decarboxylase 5 coding gene *Pdc5*, phenylpyruvate decarboxylase coding gene *Aro10*, and 3-deoxy-7-phosphoheptulonate synthase coding gene *Aro3* to block the competing pathway for aromatic amino acids can enhance the supply of precursors for the synthesis of aromatic compounds in *S. cerevisiae* (Figure 6c) [1]. Similarly, 3-deoxy-D-arabino-heptulosonate-7-phosphate synthase is subject to negative feedback regulation by Phe, Tyr, and Trp in *E. coli* and *B. subtilis*, while shikimate dehydrogenase and EPSPS catalyze the rate-limiting steps. The overexpression of these enzymes either individually or in conjunction with the other enzymes can partially alleviate these limitations [61]. In *B. subtilis*, isochorismate is synthesized by the *dhbACEBF* operon to generate (2S, 3S)-2,3-dihydro-2,3-dihydroxybenzoate (DHDHB). Therefore, generating a knockout of the gene *dhbB*, which catalyzes the first step of the DHB synthesis pathway, can increase the flux to the product menadione [61].

While increasing the flux of precursors, the participation of cofactors in the reactions catalyzed by the endogenous or heterologous enzymes also needs to be considered. For example, the reaction catalyzed by HMGR requires two molecules of NADPH as a cofactor. The overexpression of truncated HMG1 gene *tHMG1* may lead to intracellular redox imbalance and cause cell metabolic burden, affecting cell growth and product production of PACs. Common strategies for regulating the balance of NADPH cofactors include the overexpression of the mitochondrial reduced NADH kinase POS5, glucose-6-phosphate dehydrogenase ZWF1 (first NADPH-generating enzyme of the pentose phosphate (PP) pathway), transcription factor STB5 (regulating genes of the PP pathway), and YMR315W (whose promoter contains STB5 binding site) (Figure 6a) [70].

The transformation of metabolic pathways involves changes not just at the level of individual enzymes, but also in the maintenance of balance between multiple pathways, which ultimately affects the growth of cells. Static regulation of pathways plays an important role in the determination of metabolic flux; however, it cannot sense cellular conditions or promptly adjust pathway flux based on the sensed information. By contrast, dynamic regulation allows the adaptation of flux to compensate for changing conditions such as fluctuations in the temperature, nutrient availability or pH; in turn, this facilitates the delivery of the required metabolic intermediates at appropriate concentrations and times, thereby balancing cell growth and product production [117]. Based on the response to changing conditions, dynamic regulation can be categorized into metabolite-specific environmental signals, quorum sensing (QS), and gene expression-level responses (Figure 6d) [1]. Shen et al. used Gal4M9, a temperature-sensitive promoter, to design a cold shock–triggered temperature control system that separates cell growth from product production based on changes in the environmental temperature, enabling high cell-density fermentation using *S. cerevisiae* [70]. Cui et al. employed Phr60-Rap60-Spo0A, a dual-function QS system, to achieve dynamic regulation of MK-7 synthesis in *B. subtilis* strain 168, resulting in a 40-fold increase in MK-7 production in shake flask cultures [108]. Combining multiple metabolic engineering strategies for the biosynthesis of PACs can effectively enhance the production capacity of the biosynthetic pathway and improve yields, which lays the foundation for the use of microbial biosynthesis to achieve industrial production of PACs.

#### 4.3.2. Improving Reaction Efficiency Using a Multienzyme Assembly Strategy

Numerous studies have reported the use of linkers or protein scaffolds for achieving spatial colocalization of enzymes, utilizing substrate channeling to increase reaction efficiency and reduce leakage of metabolic intermediates (Table 3). This approach minimizes the carbon flux toward competing pathways and reduces the toxicity of intermediates to living cells (Figure 6f) [118]. Additionally, a recent report revealed a remarkable (>110-fold) improvement in nerolidol production after enzyme fusion; however, this is not attributable to substrate channeling after enzyme colocalization that enhances their catalytic activities, but rather, to the improvements in the expression and stability of the fusion protein [119]. For the synthesis of PACs, Han et al. initially utilized the short protein ligand GGGGS_3_ to directly fuse HGGT and truncated tocopherol cyclase (tTC), thereby promoting the biosynthesis of δ-tocotrienol. Subsequently, a pair of short peptides (regular interaction-inducing amphiphilic double helix (RIAD) and regular interaction-inducing double helix (RIDD)) were employed for scaffold-free assembly or artificial scaffolds (SH_3_, PDZ, and GBD (GTPase-binding domain)) were constructed for the assembly of HPPD and HGGT-linker-tTC, further enhancing the efficiency of δ-tocotrienol synthesis. Ultimately, the HPPD–SH_3_ ligand/SH_3_ domain–HGGT–GGGGS_3_–tTC system was selected for the construction of a substrate channel, resulting in a 156% increase in the yield of δ-tocotrienol [120].

#### 4.3.3. Engineering Modifications for Product Efflux Processes

The prenyl chain enhances the lipophilicity of aromatic compounds. The hydrophobicity of lipophilic compounds contributes to their tendency to accumulate in cell membranes. However, the limited capacity of cell membranes inherently restricts the maximization of the yield of these compounds. Moreover, their excessive accumulation in membrane compartments can induce cytotoxicity, disrupting normal membrane functions. Consequently, the intracellular storage of these products not only limits their own production, but also imposes growth stress on the cells [121]. The use of membrane engineering approaches for modifying cell membrane structures, including modifications of cell membrane composition and structure, for enhancing membrane flexibility and creating artificial storage compartments or increasing the number of transporters can promote the efflux of products (Table 3). The inclusion of extractants in the cell culture medium to facilitate the export of products from the cells can also promote the secretion of PACs and simplify extract processing (Figure 6e). Dodecane is widely used as an extractant for the in situ extraction of various terpenoids [122]. Moreover, vegetable oils such as olive and sunflower oils can be employed as alternative extractants for biphasic extraction [102]. Jiao et al. utilized 5% (*V*/*V*) olive oil for in situ extraction, enabling the extracellular secretion of 56.12% of the produced tocotrienols, thereby reducing cellular stress while enhancing tocotrienol production [102].

The secretion of PACs during biphasic fermentation potentially indicates the presence of endogenous transporters for their export. However, specific transporters of PACs have hitherto not been identified. ATP-binding cassette (ABC) transporters have been utilized for the efflux of a variety of terpenoids, including lipophilic carotenoids [123]. Therefore, ABC transporters may be utilized for the efflux of PACs. Concomitantly, the secretion of tocotrienol in the organic phase can be increased to 73.66% with the overexpression the genes of PDR11p (an ABC multidrug transporter) and YOL075c (an uncharacterized protein predicted to enable ATPase-coupled transmembrane transporter activity) [102]. In addition, the encapsulating agent cyclodextrin has been used for the transmembrane transport of lipophilic compounds due to its hydrophobic inner cavity and hydrophilic outer wall; this approach aided the extracellular secretion of 27.4% of the produced δ-tocotrienol. Moreover, the inclusion complexes formed by cyclodextrins enhance the stability of δ-tocotrienol, reducing oxidative losses [103].

The yield of membrane-bound menaquinone has been shown to be influenced by the characteristics of the cell membrane, including the content of total, saturated, and unsaturated fatty acids [21,124]. Gao et al. achieved enhanced production of menaquinone in *B. subtilis* through membrane engineering approaches involving the overexpression of AlMgs (1,2-diacylglycerol 3-glucosyltransferase, for membrane curvature), knock out of the gene nlpI (encoding lipoprotein NlpI, which increases available membrane storage by promoting Lpp-PG cross-linking), and replacement of the native promoter of FadR (a global regulator involved in lipid and fatty acid metabolism) with a strong promoter. Among these, the most effective strategy was the knockout of nlpI, which increased MK-7 production by 26.6% [95]. Furthermore, since ABC transporters consumes ATP to function, the efflux of products may compete with other ATP-dependent cellular activities. When modifying microbial hosts, it is essential to systematically design the compatibility of upstream and downstream pathways. For instance, selecting precursor supply pathways with lower ATP consumption when using metabolic engineering strategies that overexpress ABC transporters to enhance the efflux of products, such as the MVA pathway instead of the MEP pathway, should be considered.

## 5. Conclusions and Future Perspectives

### 5.1. Future Perspectives

Numerous PACs are present in plants and exhibit antioxidant, anti-inflammatory and anticancer activities, besides aiding in the prevention of neurodegenerative diseases. The synthesis of these compounds in microorganisms using synthetic biology and metabolic engineering approaches has shown significant promise. However, the complex structures of these compounds and the intricate metabolic pathways involved in their microbial production have ensured that achieving industrial-scale production of PACs using biological platforms remains a distant prospect. The MVA/MEP and shikimate pathways involved in the synthesis of the PAC precursors have been extensively studied, and the use of synthetic biology and metabolic engineering methods has yielded remarkable results. For example, metabolic engineering of *E. coli* DH5α has allowed geraniol production with yields of 131.9 g/L in a 10 L bioreactor [125]. Similarly, metabolic engineering of *S. cerevisiae* has allowed the production of 6.3 g/L of 2-phenylethanol in a 5 L bioreactor [126]. Therefore, the biosynthesis of PACs is chiefly restricted by the key enzyme (PTs).

Protein engineering approaches can be employed to modify the limited catalytic activity of PTs. With the emergence of structural biology and omics approaches as well as improvements in computing power, protein engineering has developed from directed evolution to rational and de novo design [93]. Furthermore, machine learning (ML) has emerged to overcome the cumbersome and inefficient nature of traditional directed evolution. ML employs models, such as Convolutional Neural Networks (CNNs) and Recurrent Neural Networks (RNNs), to represent the functional relationships between protein sequences and their functions [127,128]. This facilitates the identification of highly adapted sequences in a more efficient manner, thereby reducing experimental screening and enhancing mutation efficiency. Furthermore, the integration of ML with active learning creates a design–test–learn cycle that iteratively trains ML models. This approach significantly alleviates the screening burden and proves particularly effective for protein mutations lacking established techniques for high-throughput screening (HTS) [129]. Consequently, leveraging ML in conjunction with active learning can substantially reduce the burden of PT mutagenesis, enabling more rapid and effective enhancement of the catalytic activity of PTs and alteration of their substrate selectivity.

The key challenge for this method lies in the efficient sampling and utilization of information-rich protein mutants for model training. Recently, Zhang et al. developed PLMeAE, an automated protein evolution platform [130]. This platform tightly integrates a protein language model (PLM) for mutant design with biofoundry facilities for automated construction of protein variants and their testing, significantly enhancing the speed and accuracy of protein evolution. The PLM employed by this method is trained on vast datasets of natural proteins. It learns and leverages the inherent rules governing protein sequence–structure–function relationships that have been optimized through natural evolution for stability, function, and efficiency. This enables zero-shot optimization of specific proteins, a capability unattainable using conventional ML approaches [130].

Protein engineering for enzyme modification typically relies on HTS methodologies. However, the development of suitable HTS methods for PTs is hindered by the lack of appropriate physicochemical properties of the catalytic substrates amenable to standard HTS detection. Nevertheless, various automated setups have been developed for accelerating chromatography-, spectroscopy-, and mass spectrometry–based analyses [131]. For instance, Matrix-Assisted Laser Desorption/Ionization Mass Spectrometry has been utilized for screening yeast colonies at a rate of 5 s colony^−1^, enabling the rapid identification of mutant strains with improved Medium-Chain Fatty Acid production [132]. Therefore, HTS based on spectrometry can be applied for the engineering and screening of PTs to improve the efficiency of mutant selection.

The biosynthesis of PACs in microorganisms utilizes an extended enzymatic pathway. A further enhancement in production yields may be achieved by optimizing the expression of rate-limiting enzymes via extensive heterologous gene expression and precise metabolic flux engineering. The integration of heterologous and optimized gene expression cassettes at different genomic loci of *S. cerevisiae* leads to position-dependent variations in both gene expression and metabolic balance [133]. Therefore, large fragment integration to achieve coordinated and stable expression of multiple genes at consistent levels represents a critical strategy in host cell engineering.

The common host systems currently employed for large fragment integration include *E. coli*, *S. cerevisiae*, and *B. subtilis*. Established methods of integration such as lambda red recombination and related systems, the Cre–loxP system (CRAGE technology), YeastFab assembly, RADOM (Rapid Assembly of DNA Overlapping Multiple fragments, SwAP-In, the *B. subtilis* Genome Manipulation (BGM) vector system, and their combinations with CRISPR/Cas systems have been widely adopted [134,135]. Recently, Xu et al. reported a CRISPR/Cas9–mediated large DNA fragment integration (CILF) method, which employs a fusion protein (Cas9-Brex27-FadR) to target donor plasmids to DNA double-strand breaks (DSBs) while recruiting Rad51 to enhance the efficiency of homologous recombination. This study demonstrated that the presence of 1000 bp homology arms and 12 FadR binding sites on the donor plasmids allowed DNA integration with efficiencies of 93% for 10 kb fragments and nearly 80% for 40 kb fragments [136].

As a eukaryotic expression system, yeast continues to present advantages over *E. coli* and *B. subtilis* for the production of PACs [137]. The model yeast *S. cerevisiae* presents the advantages of rapid growth and genetic tractability; however, its disadvantages include limitations associated with high cell-density fermentation, stress tolerance, and substrate utilization [137,138]. By contrast, nonconventional yeasts such as *Yarrowia lipolytica*, *Pichia pastoris*, *Kluyveromyces marxianus*, *Rhodotorula toruloides*, and *Hansenula polymorpha* possess unique physiological traits. *Y. lipolytica* contains multiple pathways of intracellular acetyl-CoA synthesis, providing ample precursors for the synthesis of various products along with robust environmental tolerance (for instance, high temperature and low pH) and a broad substrate utilization spectrum. Additionally, *Y. lipolytica* can utilize low-cost substrates such as waste industrial oils, while *P. pastoris* and *H. polymorpha* can utilize methanol for growth [138].

Nonconventional yeasts have been applied for the biosynthesis of various natural products. The advancement of the CRISPR/Cas9 technology has allowed breakthroughs in genetic editing techniques in these organisms. Chen et al. achieved one-step multiplexed genome editing in *P. pastoris* by optimizing guide RNA, demonstrating that the CRISPR/Cas9 system with the HH-sgRNA-HDV (Hammerhead ribozyme at the 5′-end, small guide RNA in the middle, and hepatitis delta virus ribozyme at the 3′-end, HgH) structure allowed single-gene knock out with 95.8% efficiency. Additionally, the use of double HgH enabled one-step double-gene disruption and multigene integration, with dual-site knock out efficiency ranging from 60% to 100% and double neutral/site knock out efficiency reaching 100% [139]. Jiang et al. described TUNEYALI, a CRISPR/Cas9-based high-throughput method of metabolic engineering in *P. pastoris*, which allows adjustment of gene expression levels by replacing target gene promoters to accelerate strain development for industrial biotechnology applications and facilitate functional genomics studies. A TUNEYALI-TF library targeting 56 transcription factors (TFs) allowed enhancement in betaine production, improvement in thermotolerance, and modification of the morphological phenotypes of *P. pastoris*. The study allowed the identification of several TFs that increased thermotolerance, two TFs that eliminated pseudohyphal growth, and multiple TFs that boosted betaine production [140]. Koh et al. developed the RT-EZ (Rhodotorula toruloides Efficient Zipper) toolkit based on Golden Gate assembly, providing *R. toruloides* with optimized genetic components and streamlined procedures for multigene expression. This effectively addressed the limitations in genetic manipulation arising from its high GC content and lack of episomal plasmids [141]. The development of gene editing technologies has provided powerful tools for the high-yield production of isoprenoid aromatic compounds in nonconventional yeasts, enabling heterologous pathway expression in yeast in a more efficient manner.

Significant research has been dedicated to the heterologous production of PACs and the metabolic engineering of their biosynthetic pathways. However, the yield of PACs remains limited by several factors, including low enzymatic expression and activity, imbalanced metabolic pathways, and the suboptimal internal environment of the heterologous host. Moreover, due to the inherent complexity of living systems, the combination of individual biological components does not necessarily generate a system with the expected behavior even when the functions of them are known. With advances in computer science and AI, synthetic biology is transitioning from traditional manual trial-and-error and iterative optimization to an era of rational design based on quantitative analysis of biological systems. The major challenges in the heterologous production of PACs can be addressed through the following strategies: 1. AI-assisted modification or even de novo design of key enzymes such as PT with enhanced catalytic activity; 2. Application of flux balance analysis and constraint-based modeling methods (e.g., COBRA) [142,143], to identify optimal flux distributions that maximize target product synthesis under defined metabolic network and substrate uptake constraints, thereby balancing the metabolic flux between the MEP/MVA pathways (for prenyl donors) and the shikimate pathway (for aromatic acceptors); and 3. Exploration of non-conventional microbial chassis such as *P. pastoris* [144,145], *Ogataea polymorpha* [146,147,148], and *Rhodobacter sphaeroides* [149,150], which may offer more favorable precursor pools and catalytic environments for efficient PACs production.

### 5.2. Conclusions

Although complete heterologous synthesis of certain PACs has been achieved, a considerable number of these compounds remain to be synthesized due to constraints by various factors. Furthermore, the path toward achieving industrial-scale production of such heterologously synthesized compounds remains long. This review concisely outlines the bioactivities and biosynthetic pathways of PACs, with a primary focus on engineering strategies for their heterologous production in microorganisms. These strategies include the modification of the key enzyme PT, the selection of hosts, and diverse metabolic engineering approaches for pathways optimization. Finally, future perspectives for further enhancing PAC production through advanced engineering efforts are discussed. This paper provides a theoretical foundation for future research in this field.

## Figures and Tables

**Table 2:** PTs Catalyzing Prenylation Reactions in Microorganisms.

Enzymes	NCBI Accession NO.	Type	Source	Aromatic Compound Receptors	Prenyl Side Chain Donors	Product	Reference
SyHPT	BAA17774	UbiA	*Synechocystis* sp. PCC 6803	homogentisic acid (HGA)	GGPP	δ-tocotrienol	[71]
SfN8DT-1	BAG12671.1	UbiA	*Sophora flavescens*	naringenin	DMAPP	8-prenylnaringenin	[62]
SfFPT	AHA36633.1	UbiA	*S.flavescens*	naringenin	DMAPP	8-prenylnaringenin	[72]
AnaPT	EAW16181	DMATS	*Neosartorya fischeri*	naringenin	DMAPP	3’-prenylnaringenin	[63]
ShFPT	QXP40533.1	ABBA	*Streptomyces* sp. NT11	naringenin	DMAPP	6-prenylnaringenin	[73]
CloQ	WP_023545098.1	ABBA	*Streptomyces roseochromogenes*	naringenin	DMAPP	3’-prenylnaringenin, 6-prenylnaringenin	[74]
HlPT1L	AJD80254.1	UbiA	*Humulus lupulus*	Naringenin Chalcone	DMAPP	Desmethylxanthohumol	[75]
EsPT2	QKO29233.1	UbiA	*Epimedium sagittatum*	kaempferol	DMAPP	8-prenylkaempferol	[31]
EkF8PT	QXN66318.1	UbiA	*Epimedium koreanum*	kaempferol	DMAPP	8-prenylkaempferol	[76]
EkF8DT3			*Epimedium koreanum*	kaempferol	DMAPP	8-prenylkaempferol	[77]
XimB	AGY49248.1	UbiA	*Streptomyces xiamenensis* 318	4-hydroxybenzoate	GPP	3-geranyl-4-hydroxybenzoic acid	[78]
AePGT	ABD59796.2	UbiA	*Arnebia euchroma*	4-hydroxybenzoate	GPP	3-geranyl-4-hydroxybenzoic acid	[79]
AePGT4	ANC67957.1						
AePGT6	ANC67959.1						
iacE	A0A1J0HSL6.1	DMATS	*Pestalotiopsis fici*	resveratrol	DMAPP	2-C-prenyl resveratrol	[80]
AmbP1	AHB62774.1	ABBA	*Fischerella ambigua*	resveratrol	GPP	4-C-geranyl resveratrol,3-O-geranyl resveratrol	[26]
PcPT	BAO31627.1	UbiA	*Petroselinum crispum*	umbelliferone	DMAPP	demethylsuberosin	[81]
PsPT1	AJW31563.1	UbiA	*Pastinaca sativa*				
PpPT1	WIL06374.1		*Peucedanum praeruptorum*				
AcPT1	BBG56301.1	UbiA	*Artemisia capillaris*	p-coumaric acid	DMAPP	Drupanin and artepillin C	[82]
PcPT07	PQ310576	UbiA	*Psoralea corylifolia*	p-coumaric acid	GPP	bakuchiol	[83]
EcUbiA	BAB38446	UbiA	*Escherichia coli*	4-hydroxybenzoic acid (4-HBA)	Decaprenyl diphosphate	Coenzyme Q10	[84]
ScCoq2	P32378	UbiA	*Saccharomyces cerevisiae*	4-hydroxybenzoic acid (4-HBA)	Decaprenyl diphosphate	Coenzyme Q10	[84]
BsMenA	P39582	UbiA	*Bacillus subtilis*	DHNA	HPP	Menaquinone-7	[84]
EcMenA	P32166	UbiA	*E. coli*	DHNA	HPP, OPP	Menaquinone-7, Menaquinone-8	[85,86]
NovQ	AAF67510	ABBA	*Streptomyces niveus*	menadione	DMAPP	Menaquinone-1`	[87]
SyMenA	BAA18030.1	UbiA	*Synechocystis* sp. PCC 6803	DHNA	FPP	Menaquinone-4	[60]

## Abbreviations

PACs: prenylated aromatic compounds; 8-PN: 8-prenylnaringenin; MEP: 2-C-methyl-D-erythritol-4-phosphate; MVA: mevalonic acid; PTs: prenyltransferases; ICA: Icariin; ER: endoplasmic reticulum; PERK/eIF2α: protein kinase R -like endoplasmic reticulum kinase/eukaryotic initiation factor-2α; PTP1B: protein-tyrosine phosphatase1B; DPPH: 1,1-Diphenyl-2-picrylhydrazyl; IC50: half maximal inhibitory concentration; Ery: erysubin F; XO: xanthine oxidase; iNOS: inducible nitric oxide synthase; ROS: reactive oxygen species; RNS: reactive nitrogen species; LOO·: lipid peroxyl radicals; HCC: hepatocellular carcinoma; CDK2: cyclin-dependent kinase 2; Skp2: S-phase kinase-associated protein 2; IBD: inflammatory bowel disease; OA: osteoarthritis; TNF-α: tumor necrosis factor-α; IL: Interleukin; NO: nitric oxide; NF-κB: nuclear factor kappa–light chain enhancer of activated B cells; COX-2: cyclooxygenase; SARS-CoV-2: Severe Acute Respiratory Syndrome Coronavirus 2; PEP: phosphoenolpyruvate; E4P: erythrose 4-phosphate; DAHP: 3-deoxy-D-arabino-heptulosonate 7-phosphate; DHQ: 3-dehydroquinate; DHS: 3-dehydroshikimate; SHIK: shikimate; S3P: shikimate-3-phosphate; EPSP: 5-enolpyruvate shikimate-3-phosphate; CHA: chorismite; IPP: Isopentenyl diphosphate; DMAPP: dimethylallyl diphosphate; PYR: pyruvate; G3P: 3-phosphate; DXS: 1-deoxy-D-xylulose-5-phosphate synthase; DXP: 1-deoxy-D-xylulose-5-phosphate; DXR: 1-deoxy-D-xylulose-5-phosphate reductoisomerase; CD-ME: 4-(cytidine 5’-diphospho)-2-C-methyl-D-erythritol; MCT: 2-C-methyl-D-erythritol 4-phosphate cytidylyltransferase; HMBPP: 1-hydroxy-2-methyl-2-butenyl 4-diphosphate; CMK: 4-diphosphocytidyl-2-C-methyl-D-erythritol kinase; MDS: 2-C-methyl-D-erythritol 2, 4-cyclodiphosphate synthase; HDS: 1-hydroxy-2-methyl-2-butenyl 4-diphosphate synthase; HDR: 4-hydroxy-3-methylbutenyl diphosphate reductase; acetyl-CoA: acetyl-coenzyme A; AACT: acetyl-CoA acetyltransferase; HMGS: 3-hydroxy-3-methylglutaryl-CoA synthase; HMG-CoA: 3-hydroxy-3-methylglutaryl-CoA; HMGR: 3-hydroxy-3-methylglutaryl-CoA reductase; MK: mevalonate kinase; PMK: phosphomevalonate kinase; MVD: mevalonate diphosphate decarboxylase; IDI: isopentenyl diphosphate isomerase; NAD(P)H: nicotinamide adenine dinucleotide phosphate; GPP: geranyl pyrophosphate; FPP: farnesyl pyrophosphate; GGPP: geranyl geranyl pyrophosphate; HPT: homogentisate phytyltransferase; HGA: homogentisic acid; HGGT: homogentisate geranylgeranyltransferase; DHNA: 1,4-dihydroxy-2-naphthoate; MenA: heptaprenyltransferase; MK: menaquinone; DMATS: dimethylallyl tryptophan synthase; ABBA: α-β-β-α barrel isopentenyl transferase; 8-KAE: 8-prenylkaempferol; GST: glutathione S-transferase; SUMO: small ubiquitin-like modifier; NusA: N-utilizing protein; AI: artificial intelligence; MenG: methyltransferase; HMG1: 3-hydroxy-3-methyl-glutaryl-coenzyme A (HMG-CoA) reductase; tHMG1: truncated HMG1; MPBQMT: 2-methyl-6-phytylbenzoquinol methyltransferase; TC: tocopherol cyclase; γ-TMT: γ-tocopherol methyltransferase; Aro3/4: 3-deoxy-D-arabino-heptulosonate-7-phosphate synthase; Aro10: phenylpyruvate decarboxylase; Aro7: chorismate mutase; TyrC: cyclohexadienyl dehydrogenase; CrtE: geranylgeranyl diphosphate synthase; CrtE03M: mutant of GGPP synthase; Tkl1: transketolase 1; MOT3: transcriptional activator; POS5: NADH kinase; PDR11: ATP-dependent permease; Yol075c: ABC transporter ATP-binding protein; HPPD: 4-hydroxyphenylpyruvate dioxygenase; 2-HP-β-Cyclodextrin: 2-hydroxypropyl-β-cyclodextrin; TSC13: enoyl reductase; MdECR: very-long-chain enoyl-CoA reductase from *Malus domesticus*; TAL1: TAL bHLH transcription factor 1; ERG20: farnesyl pyrophosphate synthetase; HlPT1L: prenyltransferase gene; GmOMT2: methyltransferase from *G. max*; OMT4: 4’-O-methyltransferase; MTHFR: methylenetetrahydrofolate reductase; UbiC: chorismate pyruvatelyase; AtCOSY: coumarin synthase from *Arabidopsis thaliana*; PcU6DT: umbelliferone 6-dimethylallyltransferase from *P. crispum*; FcMS: marmesin synthase from *Ficus carica*; AtCPR1: CYP450 reductase 1 from *A. thaliana*; IUP: isopentenol utilization pathway; HepPPS: heterogeneous heptaprenyl pyrophosphate synthetase; MvaE: acetyl-CoA acetyltransferase/HMG-CoA reductase; Mav’s: hydroxymethylglutaryl-CoA synthase; UbiE: ubiquinone/menaquinone biosynthesis methyltransferase; YacM: 2-C-methylerythritol 4-phosphate cytidylyltransferase; YacN: 2-C-methylerythritol 2,4-cyclodiphosphate synthase; GlpD: glycerol-3-phosphate dehydrogenase; dhbB, bifunctional isochorismate lyase/aryl carrier protein gene; Phe: phenylalanine; Tyr: Tyrosine; Trp: Tryptophan; DHDHB: (2S, 3S)-2,3-dihydro-2,3-dihydroxybenzoate; DHB: 2,3-dihydroxybenzoate; ZWF1: NADPH-generating enzyme; STB5: regulating genes of the PP pathway; QS: quorum sensing; RIAD: regular interaction-inducing amphiphilic double helix; RIDD: regular interaction-inducing double helix; SH3: Src homology domain; PDZ: postsynaptic density 95 (PSD-95), discs large (Dlg) and zonula occludens-1 (ZO-1); GTPase: GTP hydrolases; GBD: GTPase-binding domain; ABC transporters: ATP-binding cassette transporters; AlMgs: 1,2-diacylglycerol 3-glucosyltransferase; ML: machine learning; CNNs: Convolutional Neural Networks; RNNs: Recurrent Neural Networks; HTS: high-throughput screening; PLMeAE : protein language model-enabled automatic evolution.
